# Beyond the womb: prenatal MRI’s prognostic abilities for morbidity and mortality in neonates with omphaloceles

**DOI:** 10.1038/s41372-025-02321-1

**Published:** 2025-05-16

**Authors:** Krishna Manohar, Fikir M. Mesfin, Jessica Belchos, Brandon P. Brown, Cameron Colgate, Lava Timsina, Joshua Brown, Rachel Tullar, Brian W. Gray

**Affiliations:** 1 Department of Surgery, Indiana University School of Medicine (IUSM), Indianapolis, IN USA; 2 The Fetal Center at Riley Children’s Health, Indianapolis, IN USA; 3 St Vincent Department of Surgery, Indianapolis, IN USA; 4 Department of Radiology and Imaging Sciences, Indiana University School of Medicine (IUSM), Indianapolis, IN USA

**Keywords:** Prognostic markers, Developmental biology

## Abstract

**Background:**

Managing omphaloceles poses challenges in prenatal consultation and perinatal care. We hypothesized that specific fetal MRI findings could predict morbidity and mortality in these patients.

**Methods:**

We analyzed fetal MRI studies demonstrating omphaloceles from 2006 to 2022 and conducted a retrospective review of medical records. Predictor variables were correlated with outcomes using univariate and multivariate analyses, and Receiver Operating Characteristic (ROC) curves were optimized with Youden’s J statistic.

**Results:**

Among 46 omphalocele patients, 89% survived to birth, with an overall mortality rate of 37%. Significant predictors of mortality included stomach/spleen herniation, severe anomalies, omphalocele-associated syndromes, membrane rupture, lower observed/expected total fetal lung volume (O/E TFLV), and increased percentage of liver herniation. The need for deferred repair correlated with liver/stomach herniation and “giant-omphalocele.” ROC analysis identified mortality cut points at O/E TFLV < 42% and liver herniation >77%, while deferred repair was indicated at liver herniation >51%.

**Conclusion:**

This study identified prenatal MRI findings associated with mortality and deferred repair, aiding in risk prediction and family counseling.

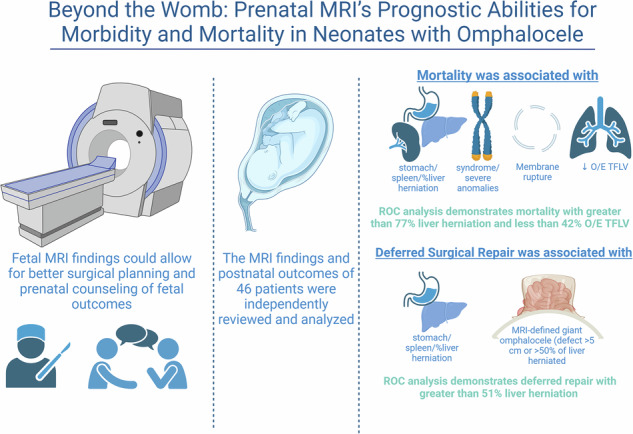

## Introduction

An omphalocele is a common congenital midline abdominal wall defect with an estimated prevalence of 0.9–2.9 per 10,000 live births [1–3]. This defect occurs when the ventral abdominal wall fails to close [4], which leads to herniation of intra-abdominal organs (such as the small bowel, colon, stomach, spleen, and liver) into a membranous sac at the base of the umbilical cord [5, 6]. Omphaloceles are frequently associated with chromosomal or syndromic anomalies including Trisomy 13, 18, and 21, Beckwith Wiedemann Syndrome, Goltz Syndrome, Omphalocele-Exstrophy-Imperforate anus-Spinal defects (OEIS) complex, and congenital heart defects [1, 4, 6, 7]. Other associated abnormalities include neural tube defects, cleft lip/palate, and limb/musculoskeletal/vertebral abnormalities [4, 7].

The ventral wall defect can vary in size and may be further classified into three categories: small, giant, and ruptured [8]. Defects less than five centimeters in diameter are considered small, those greater than five centimeters in diameter and/or containing greater than 50% liver are considered giant, and those without a membrane at birth are considered ruptured [9, 10]. Many of the latter two categories can have complex and extended hospital stays, characterized by prolonged ventilatory support and feeding issues [8, 11]. The treatment of an omphalocele depends on the size and content of the defect, with smaller defects often amenable to primary closure using the patient’s own tissues, while larger defects may require a delayed or staged repair [12]. Delayed repair encompasses utilizing techniques to allow the omphalocele membrane to form an eschar and eventually to epithelialize into a neo-skin containing sac. This is then followed by surgical closure at a later date. Staged repair involves reducing the omphalocele gradually over time through means such as a silo until the abdominal organs or use of a mesh for temporary coverage before another intervention [11–14].

Infants with omphalocele often contend with a variety of postnatal complications. Respiratory conditions include pulmonary hypoplasia, pulmonary hypertension, and post-operative respiratory insufficiency [15] and often occur as a result of a narrow chest cavity and inadequate lung growth in utero [6]. This pulmonary hypoplasia is characterized by diminished observed-to-expected total fetal lung volume ratios (O/E TFLV), lower APGAR scores, the need for a longer duration on ventilator support, and longer hospitalizations [6, 15]. Pulmonary hypertension has also been shown to be associated with the presence of liver in the hernia sac and the need for mechanical ventilation at birth [15].

Prenatal prediction of postnatal outcomes remains a challenge to the medical team caring for the fetus and child with omphalocele [6, 16]. Early detection and characterization of the omphalocele and its associated anomalies allows for proper consultation, family-centered care, and safe delivery by a multidisciplinary perinatal team. Prenatal ultrasound can detect an omphalocele as early as 12 weeks of gestation, with a reported sensitivity of between 25 and 95% [6]. However, the accuracy of ultrasound in diagnosing and delineating an omphalocele can be limited, especially in the cases of complex/giant omphaloceles and associated abnormalities [17]. In such cases, prenatal MRI has emerged as a reliable secondary diagnostic tool. MRI provides a comprehensive evaluation including: size/contents of the defect, presence of an intact membrane, specific hernia visceral contents, relationship to the umbilical cord, evaluation of cord vessels, assessing for hernia sac ascites, and evaluating fetal lung volumes [10, 16, 18]. Although the role of MRI in the management of omphalocele is not yet completely defined, some institutions routinely use it to define observed-to-expected total fetal lung volume (O/E TFLV) ratio as a potential prognostic metric [16]. However, the literature on prenatal MRI features predictive of fetal prognosis remains limited and the thresholds for better prognosis have not been determined. The aim of this study, therefore, was to evaluate findings on fetal MRI indicative of poor prognosis in a fetal omphalocele population at a single institution. We hypothesized that certain prenatal MRI markers can be used to predict morbidity and mortality in patients diagnosed with omphalocele.

## Methods

### Ethics approval

The authors have no conflicts of interest. The study was crafted and submitted for approval under our institution’s IRB process under protocol number: 17350. This study was approved as consent-exempt and HIPAA-compliant by the local Institutional Review Board and performed in accordance to the Declaration of Helsinki.

### Retrospective analysis of study population

We retrospectively identified all patients with prenatally diagnosed omphalocele who received fetal MRI at our institution between 2006 and 2022. During much of this time, fetal MRI was limited to cases identified as high risk by prenatal ultrasound at 18–20 weeks gestation or when diagnosis by ultrasound was in question. Selection of the forty-six patients was based on complete availability of clinical data and confirmation of omphalocele at birth based on retrospective chart review. Fetal MRI studies were conducted at an average gestational age of 28 weeks and usually were accompanied by basic karyotype testing and fetal echocardiography. Further genetic testing was offered to high-risk fetuses who met criteria based on the previous findings. Exclusion criteria for final analysis included multiple gestation pregnancies and patients lost to follow up due to lack of imaging data, and early intra-uterine fetal demise. There were no early terminations of pregnancy of our cohort, and mortality was assessed for the entire cohort with the remainder of the variables tested assessed for the living cohort. Imaging biomarkers identified on MRI for analysis included observed to expected total fetal lung volume (O/E TFLV), membrane rupture, defect size, and herniation of spleen, stomach, liver, and other organs (including gallbladder or bowel). Comorbidities and other anomalies found on prenatal imaging were also noted. An independent investigator completed a retrospective review of the chart for patient characteristics at birth including sex, karyotype abnormality, ECHO results (categorized as normal, mild PFO/PDA, minor abnormalities-requiring no intermediate intervention, and major abnormalities-requiring medical and/or surgical intervention), weight (g), presence of a giant omphalocele (defined as greater than 50% of liver out or greater than 5 cm in defect size), sac rupture, syndrome present (including OEIS, Pentalogy of Cantrell) and presence of scoliosis, vertebral anomalies, or limb defects). Patient outcome variables were also assessed including survival to birth, survival 24 h, overall survival, length of stay, respiratory support at 30 days (defined by usage of nasal cannula or need for mechanical ventilation), presence of pulmonary hypertension, nutritional support (defined by presence of NG or g tube feeding), and type/timing of surgical intervention received. “Deferred surgical repair” was used to describe either a delayed surgical (via granulation) or staged surgical repair (silo or mesh use) as defined above.

### MRI analysis

MRI was performed on a 3-T Skyra MR scanner (Siemens Healthineers, Erlangen, Germany). Women were positioned supine. Half-acquisition single-shot fast spin echo (SSFSE), steady state free precession (SSFP), T1 spoiled 3-D gradient, and gradient echo (GRE) images were obtained of the entire fetus in the axial, sagittal, and coronal planes. The scanning time and slice was less than 1 slice per image and the entire MRI study was completed within 45 min. No contrast media was used. MRI analysis was conducted by a subspecialty-trained pediatric radiologist with ten years of experience interpreting fetal MRI as well as a radiology trainee physician under supervision, blinded to the patient diagnosis and outcomes. Magnetic resonance studies, prior to segmentation, were reviewed to ensure adequate quality of omphalocele imaging and those with inadequate visualization (low resolution, or motion artifact) were excluded from analysis. Volumetric calculations were conducted and compared to published standards to calculate observed to expected total fetal lung volume ratio [19, 20]. The liver, stomach, spleen, and other organs were noted for herniation into omphalocele. Specifically, for quantification of liver herniation: semi-automated segmentation of the liver was performed by a single observer (R.L.W.) in the image series in which the defect and liver were best viewed (most commonly in axial plane) using Philips Intellispace ® software Tumor Tracking Tool (Koninklijke Philips N.V., Amsterdam, Netherlands). Although propagation of the region of interest (ROI) with edge detection occurred automatically through the image series, manual adjustment of the ROI was performed in each slice of the selected sequence to ensure accuracy. The volume of the ROI was calculated based on slice thickness by the software and recorded for analysis. A subset of images representing 10% of the study population were measured by a second observer (B.P.B.) to assess accuracy and interrater reliability and no substantive changes were found with this internal validation technique. Other MRI features noted include: presence of an intact membrane; whether or not the defect was considered “giant” (defined by greater than 50% of liver herniation or greater than 5 cm fascial defect); presence of any anomaly categorized as minor (such as small structural defects including umbilical cord cysts, limb defects, minor scoliosis), moderate (moderate vertebral anomalies, neural tube defects, or mild/moderate genitourinary defects), and severe (including combination of several “moderate” anomalies, severe neurologic/ cardiopulmonary anomalies, or severe vertebral anomalies); presence of OEIS or Pentalogy of Cantrell features; and presence of limb defects.

### Statistical analysis

We assessed the relationship between patient MRI characteristics and patient characteristics at birth as well as patient outcomes. Next, we looked at how well findings on MRI correlated with patient characteristics at birth using cross-tables analysis. Univariate analysis was conducted using two-sample t-tests for continuous and Fisher’s exact tests for categorical characteristics with significance defined as *p* < 0.05. We then performed a Receiver Operating Characteristic (ROC) curve analysis to correlate continuous variables such as O/E TFLV and % liver herniation in relation to mortality and need for deferred repair. Youden’s J statistic was used to optimize cutoff points on the ROC curve. We then created a binomial logistic regression model of overall mortality with significant variables from our univariate analysis and known significant variables in the literature, keeping those variables that did not violate parameters of the regression. Significant variables that were strongly associated with many other variables were excluded. Stomach herniation and whether or not the membrane was intact was strongly associated with spleen herniation and these variables were excluded. Severe anomaly type was strongly associated with “presence of a syndrome/complex” and was excluded. The model ultimately included O/E TFLV percentage, percentage of herniated liver and spleen, presence of a syndrome/complex, and MRI-defined giant omphalocele (>5 cm defect or >50% of liver herniated) to estimate the adjusted odds ratio with 95% confidence intervals of the independent effect of the above variables on overall mortality. Analyses were performed using SPSS (IBM SPSS Statistics 27).

### Data statement

Data will not be publicly published as the data contains sensitive information that would be potentially identifiable. Interested parties are welcome to reach out to authors for parts of the dataset with protected health information and identifying information removed.

## Results

### Patient cohort characteristics

**Fig. 1:**
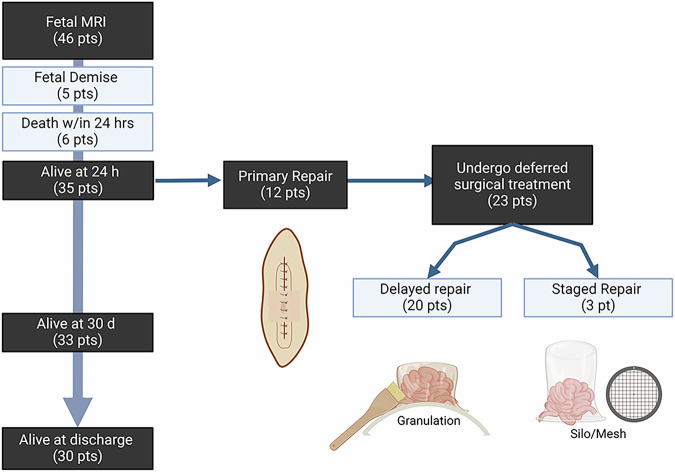
Postnatal course of patient with omphalocele. This figure displays the postnatal course of patients who underwent fetal MRI between 2006 and 2022. Six patients died at birth, with 3 dying at an outside-system hospital. An additional 5 patients passed away within 24 h. During the next month, 12 patients had a primary repair, and 23 patients underwent deferred repair. Of those, 20 had a delayed repair and 3 had a staged repair. Thirty patients were alive at discharge.

We identified a total of forty-six patients who were diagnosed with omphalocele by prenatal ultrasound and received fetal MRI at our institution between 2006 and 2022. Fetal MRI exams of sufficient quality and a minimum of four key pulse sequences (axial SSFSE, coronal SSFSE, sagittal SSFSE, and coronal T1 weighted imaging) were used to identify the patients and conduct measurements. The postnatal course of the patients with omphaloceles are illustrated in Fig. 1.

Descriptive statistics including characteristics at birth, fetal MRI features, and patient outcomes are displayed in Table 1A–C. In terms of patient characteristics at birth (Table 1A), there were slightly more females (54.3%) with a mostly normal karyotype at birth (92.5%), weighing on average 2.57 kilograms. Most had an associated echocardiogram abnormality (95%)—with 30% having a major abnormality defined as requiring surgical intervention, 30% having a minor abnormality requiring no surgical intervention, and 35% having mild PFO/PDA. Seventy-four percent were identified as having a giant omphalocele (greater than 50% of liver herniated, or fascial defect greater than 5 cm), and 20% had membrane rupture of the omphalocele sac at birth. Approximately 25% had either OEIS syndrome or Pentalogy of Cantrell features, 30% had scoliosis, 16% had vertebral anomalies, and 42% had a limb defect.

**Table 1:** Demographics, outcomes, and MRI characteristics of cohort who received fetal MRI.

(A) Patient characteristics at birth	Mean ± SD/frequency
Sex (M/F)	
*Male*	45.7% (21)
*Female*	54.3% (25)
Karyotype abnormal? (*n* = 40)	7.5% (3)
ECHO (*n* = 40)	
*No abnormality*	5% (2)
*Mild PFO/PDA*	35% (14)
*Minor abnormality*	30% (12)
*Major abnormality*	30% (12)
Weight (kg)	2.57 ± 0.65 (*n* = 44)
Giant Omphalocele	73.9% (34)
Sac ruptured?	19.6% (9)
OEIS syndrome	11.6% (5)
Pentalogy of Cantrell syndrome	14.0% (6)
Scoliosis? (*n* = 43)	30% (13)
Vertebral anomalies (*n* = 43)	16% (7)
Limb defect (*n* = 43)	42.4% (14)
**(B) Fetal MRI features (*n*** **=** 46)	**Mean** **±** **SD/Frequency**
Giant	69.6% (32)
Presence of liver in hernia sac	84.8% (39)
*Avg percentage of liver herniated*	64.63 ± 37.45%
Presence of spleen in hernia sac	23.9% (11)
Presence of stomach in hernia sac	37% (17)
Membrane intact	82.6% (38)
O/E TFLV (%)	51.7% ±31.3%
Anomaly	84.8% (39)
*Minor*	32.6% (15)
*Moderate*	19.7% (9)
*Severe*	32.6% (15)
Syndrome	32.6% (15)
*OEIS*	13% (6)
*Pentalogy of Cantrell*	10.9% (5)
Limb defect alone	23.9% (11)
**(C) Patient outcomes**	**Mean** **±** **SD/Frequency**
Mortality	
*At Birth*	10.9% (5)
*By 24* *h*	23.9% (11)
*Overall*	37.0% (17)
LoS for pts that survived to dc (days) (*n* = 30)	64.7 ± 66
Respiratory status at 30 days (*n* = 33)	
*Room air*	54.5% (18)
*Nasal canula*	24.2% (8)
*Endotracheal tube/tracheostomy*	21.2% (7)
Pulmonary hypertension at 30 days (*n* = 33)	27.3% (9)
Nutritional support at 30 days (*n* = 33)	63.6% (21)
Treatment pathway after birth (*n* = 35)	
*Primary closure*	34.3% (12)
*Deferred repair*	65.7% (23)
*Mesh/Silo*	8.6% (3)
*Granulation*	57.1% (20)

Omphalocele patients who underwent MRI (*n* = 46).

*LoS* length of Stay, *OEIS* omphalocele-exstrophy-imperforate anus-spinal defects, *O/E TFLV* observed/expected total fetal lung volume (expressed as a percentage).

Fetal MRI characteristics are displayed in Table 1B. Seventy percent of fetal MRI’s demonstrated a giant omphalocele. The liver, spleen, and stomach were herniated into the sac 85%, 24%, and 37% of the time respectively. The membrane was intact in 83% of cases and the average observed/expected total fetal lung volume ratio was 52%. On fetal MRI, an anomaly was noted in 85% of cases. Fifteen of these cases were organized into minor anomalies and included small structural defects including umbilical cord cysts, limb defects, minor scoliosis, mild ventriculomegaly. Another nine cases were organized into moderate anomalies and included: moderate vertebral anomalies, neuro/neural tube defects, or mild/moderate genitourinary defects. And finally, fifteen of the cases were categorized as severe anomalies which included a combination of several moderate anomalies, and/or severe neurologic/ cardiopulmonary anomalies, and/or severe vertebral anomalies. A syndrome/complex was suspected in 33% of cases and included: OEIS (13%), Pentalogy of Cantrell (11%), trisomies, limb-body wall defect, and prune belly.

The outcomes of these patients are displayed in Table 1C. Of the forty-six patients, 11% died at or immediately after birth (with an overall mortality of 37%). Thirty-five patients were alive at 24 h. Of these patients, twelve underwent primary repair within a few days of life, and 23 patients had deferred surgical management (with delayed repair, staged repair, or mesh placement). At one month, thirty-three patients remained alive. Of these patients, 46% required some form of respiratory support (with 21% requiring ventilator support) and 27% had pulmonary hypertension. Thirty patients were started on enteral feeds, and of these 64% required nutritional support via a gastrostomy tube or nasogastric feeds.

### Fetal MRI features are predictive of postnatally defined morphological anomalies

Several MRI variables were compared with patient characteristics at birth, and their associations are displayed in Table 2. Fetal MRI findings suggesting of OEIS/Pentalogy of Cantrell Syndromes, limb defects, omphalocele membrane defect, and presence of a giant omphalocele were strongly associated with those respective findings at birth (*p* < 0.0001).

**Table 2:** MRI predictors of patient characteristics at birth.

Patient characteristics at birth	MRI finding	*p*-value
OEIS (*n* = 5)	Suggestive of OEIS (*n* = 6)	*p* < 0.0001
Pentalogy of Cantrell (*n* = 6)	Suggestive of pentalogy (*n* = 5)	*p* < 0.0001
Limb defect (*n* = 14)	Suggestive of limb defect (*n* = 11)	*p* < 0.0001
Sac ruptured (*n* = 9)	Membrane defect (*n* = 8)	*p* < 0.0001
Giant omphalocele (*n* = 34)	Suggestive of giant omph (*n* = 32)	*p* < 0.0001

Patient characteristics at birth are shown in association with Fetal MRI findings. Cross-tabulations and chi-square statistics were performed to test the strength of the association of each pair of variables, with *p*-values displayed.

*OEIS* omphalocele-exstrophy-imperforate anus-spinal defects, *Omph* omphalocele.

### Fetal MRI imaging findings are strongly associated with patient mortality

On univariate analysis, the presence of severe anomaly (*p* = 0.033) was the only MRI variable associated with mortality at birth, defined as a stillbirth or death within a few hours of birth. The presence of stomach herniation (*p* = 0.012), spleen herniation (*p* < 0.0001), presence of severe anomalies (*p* = 0.0004), presence of a syndrome or complex (*p* = 0.048), membrane rupture (*p* = 0.038), observed to expected total fetal lung volume (O/E TFLV) (*p* = 0.019), and percentage of liver herniation (*p* = 0.013) were associated with overall mortality (Table 3).

**Table 3:** Univariate analysis of predictor variables to outcomes.

Predictor variables	Mortality at birth	Overall mortality	Deferred repair?	Respiratory support?	Nutritional support?
Liver herniation	1.000	0.234	**0.033**	0.057	0.660
Stomach herniation	0.365	**0.012**	**0.015**	0.392	**0.024**
Spleen herniation	0.128	**<0.0001**	0.275	1.000	0.503
Anomaly type (Severe)	**0.033**	**0.0004**	0.380	0.364	0.066
Limb abnormalities	0.580	0.722	0.640	0.660	0.184
Syndrome/Complex	0.311	**0.048**	0.121	0.660	0.358
Pentalogy of Cantrell	0.453	0.055	0.536	0.400	1.000
OEIS	0.520	0.655	1.000	1.000	0.255
Giant defect^a^	0.633	0.195	**<0.0001**	**0.018**	0.266
Sac ascites	0.306	1.00	0.211	0.669	0.669
Membrane intact	0.203	**0.038**	0.536	0.152	0.503
O/E TFLV	0.361	**0.019**	0.497	0.310	0.762
Liver herniated %	0.853	**0.013**	**<0.0001**	**0.006**	0.258

^a^Giant defect: >5 cm or >50% of liver herniated on MRI.

The below table shows the correlation of predictor variables to mortality at birth, mortality at discharge, need for a deferred repair, need for respiratory support, and need for nutritional support respectively.

Significance defined by p<0.05.

### Fetal MRI imaging findings are correlated with patient outcomes

Deferred repair (as defined above) is correlated with several predictors including the presence of liver (*p* = 0.033) or stomach (*p* = 0.015) within the hernia sac, percent of liver herniation (*p* < 0.0001), and MRI defined “giant omphalocele” (*p* < 0.0001). The need for respiratory support at 30 days of life was also associated with percent of liver herniation (*p* < 0.0001) and MRI defined “giant omphalocele” (*p* < 0.0001). Finally, the presence of stomach herniation was the only variable significantly correlated to the need for nutritional assistance at 30 days (Table 3).

### Optimized ROCs for fetal lung volumes and liver herniation for mortality and deferred repair

**Fig. 2:**
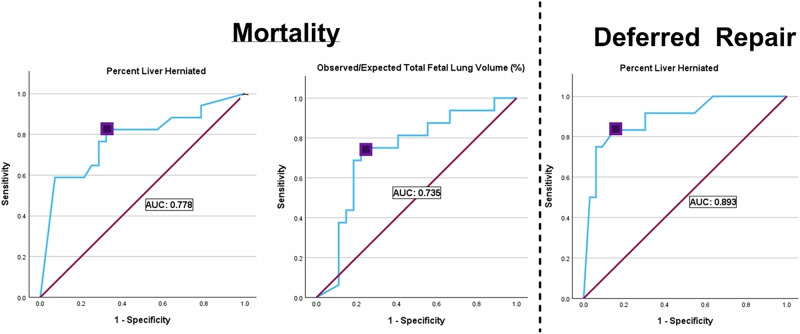
Receiver operating characteristic curves for predictors for mortality and deferred repair. Receiver operating characteristic curves were used to determine predictive cutoff points of MIR variables by using O/E TFLV ratio and % Liver Herniation for mortality and using % Liver Herniation to predict deferred repair.

Receiver operating characteristic (ROC) curve analysis was performed on continuous variables in relation to mortality and the need for deferred repair (Table 4), based on the significance found in the univariate analyses in Table 2. The cut point for observed-to-expected fetal lung volume (O/E TFLV) as a predictor for overall mortality was found to be less than 42% (*p* = 0.001) (Fig. 2). The cut point for percentage liver herniation for mortality was found to be 77% (*p* = 0.003) (Fig. 2). Taken together this indicates that fetuses with MRI findings of an OE/TFLV ratio less than 42% and herniation of the liver greater than 77% had a higher risk of mortality. When looking at continuous predictors for deferred repair, the cut point for liver herniation percentage was greater than 51% (Fig. 2) indicating that liver herniation greater than 51% was predictive of a need for a deferred repair (staged or delayed) over that of a primary repair.

**Table 4:** ROC analysis of continuous predictors on mortality and need for deferred repair.

Predictor	Outcome	Optimal cut point	Sensitivity	Specificity	Area under curve
O/E TFLV	Mortality	42%	0.750	0.778	0.735
Liver herniation %	Mortality	77%	0.824	0.680	0.778
Liver herniation %	Delayed Repair	51%	0.833	0.848	0.893

Continuous variables including variables. O/E TFLV ratio and liver herniation percentage are correlated to mortality and need for deferred repair.

### Several variables remain potential predictors of mortality on a regression model

A binomial logistic regression was employed to look at independent predictors of overall mortality. From this model, the presence of spleen herniation had a nearly 20 times greater odds of mortality. Additionally, if features of MRI were indicative of a syndrome or complex (including OEIS, Pentalogy of Cantrell, Trisomy, Prune-Belly Syndrome, Limb-Body Wall Disorder) patients were at a nearly 9 times greater odds of mortality (Table 5). The observed/expected total fetal volume ratio is also a nearly significant independent variable in this model (*p* = 0.079) and suggests that a one-unit increase in lung-volume percentage decreases the odds of mortality by a factor of 0.965 (or 3.5%). For a better scaled understanding, every 10 unit increase in lung volume percentage suggests a decrease of mortality by a factor of 0.697 (or 30.3%).

**Table 5:** Multivariate logistic regression of MRI predictors of mortality.

MRI predictor	Odds ratio	*P*-value
O/E TFLV%	**OR: 0.965 [0.927–1.004]**	***p*** = **0.079**
Spleen herniated	**OR: 20.334 [1.770–233.564]**	***p*** = **0.016**
Liver herniated %	OR: 0.998 [0.949–1.050]	*p* = 0.946
Presence of syndrome/complex	**OR: 8.784 [1.136–67.948]**	***p*** = **0.037**
Giant (>5 cm, >50% of liver)	OR: 1.916 [0.043–84.596]	*p* = 0.737

Binomial logistic regression of MRI predictors of mortality.

Significant variables for overall mortality in binomial logistic regression showing that spleen herniation and presence of a syndrome/complex are independent predictors of mortality and that O/E TFLV ratio % is trending towards being an independent predictor of mortality.

Significance defined by p < 0.05.

## Discussion

Omphalocele is a congenital ventral wall defect that can lead to significant morbidity and mortality in the affected infants. Early and accurate diagnosis is crucial to not only improve prenatal counseling, but also to improve outcomes for these patients by helping to coordinate a multi-disciplinary team [11] that may result in better postnatal management. Although giant and ruptured omphaloceles have statistically worse outcomes [6, 21, 22], the variability amongst cases of omphaloceles makes categorization of cases into traditional diagnostic criteria difficult, as many cases fall between two or more diagnostic categories [23]. Therefore, predictive prenatal biomarkers may be a better option for designing decision algorithms and use in advising patient families.

Ultrasound is the primary method of screening for omphalocele and can be performed as early as 12 weeks, however the sensitivity is variable, ranging from 25 to 95% [6]. Previous institutions have attempted to use ultrasound to predict outcomes as well. Montero et al demonstrated that omphalocele diameter to head circumference (OD/HC) greater than 0.21 was a good predictor of need for deferred repair, however sensitivity and specificity of this predictor were 84.6% and 58.3% respectively [24]. Another group correlated the use of omphalocele circumference/abdominal circumference (OC/AC) ratio for predicting the herniation of liver and type of surgical reconstruction in a cohort of 21 [6, 25], while an omphalocele diameter to abdominal diameter (OD/AD) greater than 0.8 and liver herniation were also associated with increased morbidity [6, 26]. The presence of liver herniation was also used as a marker of adverse outcomes, with an AUC of 0.74 [27], however used a much smaller cohort. Although an excellent tool for diagnosis as demonstrated above, MRI can be an excellent adjunct for offering more points data to make difficult decisions regarding more complex cases of omphalocele. Our study of forty-six cases of prenatally diagnosed omphalocele demonstrates the potential use of fetal MRI imaging markers for fetal prognostication and surgical planning.

The role of fetal MRI was initially found to be most helpful in predicting fetal lung volumes in determining morbidity and mortality from respiratory insufficiency from pulmonary hypoplasia [6, 15, 28]. Correlations between pulmonary hypoplasia and worse outcomes are consistent with findings of other single omphalocele cohorts highlighting the importance of the metric for providers [16, 29, 30]. Overall, our data emphasize this importance and demonstrate that a reduced O/E TFLV is a potential independent predictor of mortality in a bivariate regression and suggest a cut-off point of less than 42% O/E TFLV as an indicative of a higher risk of mortality. This is in line with work by Danzer et al, which demonstrated the importance of O/E TFLV ratio in neonatal outcomes—showing that an increase in O/E TFLV from 25% (severe pulmonary hypoplasia) to 50% (moderate), to (>50%) showed significantly increasing survival at 60%, 92%, and 96% [8, 16]. Additionally, work by Dadoun et al. similarly illustrates the importance of this measure and found that in their cohort of patients, an O/E TFLV of less than 50% was associated with mortality, need for intubation, and pulmonary hypertension.

Liver herniation is a known poor prognostic factor in the management of omphalocele, however our data indicate that the percent of liver herniated might be a more specific and useful metric. Some investigations have used the presence of liver herniation as a marker of adverse outcomes in a small cohort, for both cases of omphalocele and gastroschisis [27]. In our cohort, the presence of liver herniation alone was only correlated with the need for deferred repair. We were able to use the percentage of liver herniated into the sac to create optimum cutoff points for mortality (greater than 77%) and to predict the need for deferred repair (greater than 51%). In our combined bivariate regression model however, percentage of liver herniated was not a significant independent predictor of mortality. But, our data and the literature still indicate the usefulness of this measure to providing guidance on postnatal management and surgical planning, such as the need of cesarean delivery [6] to prevent liver injury.

Although other organ herniation has been used, our cohort data is novel as it shows the importance of using stomach and spleen herniation to provide additional metrics of analysis for prognosis. Spleen herniation was correlated to overall mortality, while stomach herniation was correlated with overall mortality, deferred repair, and need for nutritional support. Stomach herniation was not able to be used in the regression model as it was too linearly correlated with other predictor variables including spleen herniation. Spleen herniation, however, remained a strong independent predictor of mortality in the regression model.

And finally, MRI is also able to reliably characterize anomalies that help define a fetal syndrome or complex. We saw that MRI findings characteristic of OEIS, Pentalogy of Cantrell, and limb defects were strongly correlated with those same associated characteristics at birth (*p* < 0.0001). An interesting point of future study would be how the use of MRI compares with ultrasound in the identification of these anomalies. In our regression model, we used the definition of syndrome/complex as including the following diagnoses in our cohort: OEIS, Pentalogy of Cantrell, trisomies, limb-body wall defect, and prune belly. The presence of these syndromes/complexes were also a significant independent predictor of mortality.

## Limitations

Despite our findings, our study was limited by the size of the cohort and single-center population making it vulnerable to selection bias, confounding factors, and lack of statistical power. In addition, some patients were lost to follow up. And finally, our study spans 16 years which likely shows an improvement in MRI technology and neonatal outcomes near the latter half of the study. Our sample selection is too small to stratify the difference in health outcomes of MRI technology improvements, but this could be an interesting target of a future study. Our initial selection of patients is limited as a retrospective identification of patients identified to have an omphalocele on MRI. These high-risk patients include a potential identification of omphalocele or any other birth defect on ultrasound, multiple gestation, growth restriction, and more. Therefore, we were not able to capture smaller omphaloceles that may have been missed on ultrasound or were in lower-risk patients. In addition, we were unable to capture patients who were born to mothers that did not receive adequate prenatal care due to a variety of health care disparities. Therefore, we have a selection bias in our initial cohort toward more high-risk patients able to access healthcare. However, our analysis and conclusions are still relevant to this population and could be extrapolated to other groups. Future studies should be multi-institutional and conducted on larger populations to increase the power and generalizability of our study. In doing so, we believe that by using weighted and normalized coefficients from logistic regression of mortality a mortality prediction calculator can be created and employed during prenatal consultation. In addition, more granularities can be provided for surgical planning.

## Conclusion

Fetal MRI is emerging as a new and useful tool in the management of prenatally diagnosed omphalocele. By providing detailed information on the extent of organ herniation, percent liver herniated, identification of other anomalies, and determination of fetal lung volumes, fetal MRI allows for more prognostic information to be provided to parents and healthcare providers. More specifically, this information can assist in decisions regarding preterm delivery, mode of delivery, and timing of surgery, ultimately leading to improved outcomes for affected infants. Additionally, fetal MRI can help in identifying associated anomalies and guide further medical evaluations, which can significantly impact the management and treatment of affected infants. Therefore, incorporating fetal MRI as a prognostic tool in the management of larger and more complex omphaloceles can be a crucial component of prenatal care and can significantly improve the prognosis, enhance patient-family consultation, and help clarify postnatal surgical management. Future directions include developing a multi-institutional study to increase power and improve our predictive model as well as to further clarify the role of MRI in the diagnosis and management of fetal omphalocele.
